# Climate Is Not All: Evidence From Phylogeography of *Rhodiola fastigiata* (Crassulaceae) and Comparison to Its Closest Relatives

**DOI:** 10.3389/fpls.2018.00462

**Published:** 2018-04-10

**Authors:** Jian-Qiang Zhang, Da-Lv Zhong, Wei-Jie Song, Ruo-Wei Zhu, Wei-Yue Sun

**Affiliations:** College of Life Sciences, Shaanxi Normal University, Xi’an, China

**Keywords:** Crassulaceae, Hengduan Mountains, Qinghai-Tibetan Plateau, Quaternary climatic oscillations, *Rhodiola fastigiata*

## Abstract

How geological events and climate oscillations in the Pleistocene glaciation shaped the geographic distribution of genetic variation of species on the Qinghai-Tibetan Plateau (QTP) and its adjacent areas has been extensively studied. However, little studies have investigated whether closely related species in the same genus with similar physiological and life history traits responded similarly to the glacial climatic oscillations. If this is not the case, we would expect that the population histories of studied species were not driven by extrinsic environmental changes alone. Here we conducted a phylogeographic study of a succulent alpine plant *Rhodiola fastigiata*, using sequences from chloroplast genome and nrITS region, as well as ecological niche modeling. The results of *R. fastigiata* were compared to other congeneric species that have been studied, especially to *R. alsia* and *R. crenulata*. We found that for both markers, two geographic groups could be revealed, corresponding to the QTP plateau and the Hengduan Mountains, respectively, indicating isolated refugia in those two areas. The two groups diverged 1.23 Mya during the Pleistocene. We detected no significant population expansion by mismatch distribution analysis and Bayesian Skyline Plot. We found that even these similar species with similar physiological and life history traits have had different demographic histories in the Quaternary glacial periods. Our comparative phylogeographic study sheds new lights into phylogeographic research that extrinsic environmental changes are not the only factor that can drive population demography, and other factors, such as coevolved interactions between plants and their specialized pathogens, that probably played a role need to be examined with more case studies.

## Introduction

How geological events and climate oscillations in the Pleistocene glaciation affected the distribution of genetic variation of species over time and space is a central issue in phylogeographic studies (Avise, 2009). As more and more species are surveyed phytogeographically, scientists are interested in whether organisms that inhabit the same community or have similar distributions experienced the same phylogeographic history, which is the focus of comparative phylogeography (Bermingham and Moritz, 1998; Avise et al., 2016). These studies utilized statistical phylogeography and ecological modeling to provide important insights into how congruence and incongruence happen (e.g., Barrow et al., 2017). Recent development includes statistically comparing different hypothetical models about the structure of refugia and post-glacial re-colonization routes, as well as spatially explicit phylogeographic analyses (Lemmon and Lemmon, 2008; Barrow et al., 2015). However, less studies have considered how closely related species in the same genus or lower taxonomic group with similar physiology and life histories respond to climatic oscillations. Other studies demonstrated that species with different characters (e.g., stress tolerance, life cycle, and dispersal ability) might have experienced different glacial histories (Shafer et al., 2010; Stewart et al., 2010). Nevertheless, if species with similar physiological or life-history traits showed different demographic histories, climate and intrinsic traits are not enough to explain demographic changes of species. Thus it is of great interest to explore how species in the same genus responded to the Quaternary climatic oscillations and if other factors affected species demographic changes.

Phylogeographic studies in the Qinghai-Tibetan Plateau (QTP; including the plateau platform, Himalaya and Hengduan Mountains) have increased in recent years (Liu J.Q. et al., 2012), as this area is an important alpine biodiversity hotspot (Myers et al., 2000; Mittermeier et al., 2004) with a very complex geological history and ecological heterogeneity. Recent studies have showed complex evolutionary scenarios for species in this area and different patterns in response to the geological events and climatic oscillations (Liu J.Q. et al., 2012). It is common for plants to survive in the refugium in Hengduan Mountain area (Yang et al., 2008; Zhang et al., 2010), while there might be some other small regional refugia on the plateau platform (Wang et al., 2009a,b, 2010; Opgenoorth et al., 2010). Two recent comparative studies showed that distribution of focal species remained more or less stable during the Quaternary (Luo et al., 2016, 2017), raising the question of what attributes of plants decided their reaction to glacial climatic oscillations.

*Rhodiola*, a genus mainly distributed in the QTP area, provides us an ideal model to study how much plants from the same genus differs in glacial history. There are several species in *Rhodiola* that have been studied phylogeographically: *Rhodiola alsia* (Gao et al., 2009), *R. dumulosa* (Hou and Lou, 2014), *R. kirilowii* (Zhang et al., 2014), *R. crenulata* (Zhang et al., Unpublished) and sect. *Trifida* (Li et al., 2018). These species represent different morphological space of *Rhodiola*: dioecious or hermaphrodite, having elongated rhizomes or not, bearing persistent old flowering stems or not. Here we added an example of *R. fastigiata*, which is morphologically very similar to *R. alsia*, to see if closely related species with similar distribution and morphology responded the same to the Quaternary climatic oscillations.

*Rhodiola fastigiata* (Hook. f. & Thomson) S.H. Fu is a perennial herb with a conspicuously elongated rhizome and dense persistent flower stems. It is distributed on rocky slopes at ca. 3500–5400 m in elevation of the QTP area (Fu and Ohba, 2001). The distribution pattern of the species is island-like, as the populations are isolated by forests at lower elevation. Here we used ITS and two plastid sequences, as well as ecological niche models (ENMs) to infer demographic history of this species. We also compared the results with other congeneric species to test the hypothesis that closely related species should have similar reactions to glacial climatic oscillations as they bear the same intrinsic traits and experienced the same extrinsic environmental changes.

## Materials and Methods

### Population Sampling

Through 2016–2017, we collected 22 populations of *R. fastigiata* in Yunnan, Sichuan, and Xizang provinces, covering all its distribution range. For each population, we sampled 7–20 individuals at least 20 m apart. The details of the collected samples are shown in **Supplementary Table S1**. Fresh leaves were directly put into silica gel for quick dehydration. We included a total of 287 individuals of *R. fastigiata*. *R. coccinea* were also collected for outgroup in the analyses.

### DNA Extraction, PCR Amplification, Cloning and Sequencing

We used Plant Genomic DNA Kit (TianGen Biotech, Xi’an, China) to extract DNA from silica-gel dried leaves. ITS-1 and ITS-4 (Mayuzumi and Ohba, 2004) primers were used for amplifying ITS, c and f (Taberlet et al., 1991) for *trnL-F*, and trnS and trnG for *trnS-G* (Hamilton, 1999). Polymerase Chain Reaction (PCR) mixture were 20 μl with 2 μl 10× buffer, 0.5 μl of each primer, 0.4 μl of dNTP mixture, 1 U of Taq polymerase (TianGen Biotech, Xi’an, China) and 1 μl template genomic DNA. The PCR cycling programs followed Liu P.L.et al. (2012). The same amplification primers were used for sequencing of the chloroplast fragments and most ITS sequences. For individuals that have multiple peaks, we ligated the PCR products into pGEM-T Easy Vector using a Promega Kit (Promega Corporation, Madison, WI, United States). Then we chose plasmid that containing the right insertion to sequence. Sequencing was conducted on a 3730 automatic DNA sequencer in Tsingke Biotech, Xi’an, China. We used ContigExpress (a component of Vector NTI Suite 6.0, InforMax) to check and assemble contigs, and ClustalW v 1.7 (Thompson et al., 1994) to align the sequences. Finally, we manually checked the aligned matrix in BioEdit v 7.0.1.

### Phylogeny, Divergence and Biogeography Analysis

As chloroplast genome evolves as a whole in plant, we concatenated the two plastid sequences. We extracted unique sequences (haplotypes and ribotypes) using DnaSP v5 (Librado and Rozas, 2009). We ignored indels as the two indels in *trn*S-G only occurred in one individual, respectively. To infer phylogenetic relationships among haplotypes and ribotypes, we implemented Maximum parsimony and Bayesian inference to reconstruct phylogeny with PAUP^∗^ 4.0b10 (Swofford, 2003) and MrBayes 3.2.1 (Ronquist and Huelsenbeck, 2003), respectively. One individual of *R. coccinea* was used as outgroup according to previous study (Zhang et al., 2014). For the MP tree, we set heuristic searches with MULTREES and TBR branch swapping. One thousand replicates of random addition sequences were used to construct staring trees. Bootstrap values (Felsenstein, 1985) were applied to evaluate support for nodes. One thousand replicates with ten replicates of random addition sequence and NNI branching swapping were applied. As Bayesian method needs an appropriate substitution model, we first determined it (TPM2uf + I + G for plastid dataset, and SYM + G for the ITS dataset) with Akaike information criterion (AIC) in Modeltest 3.7 (Posada and Buckley, 2004; Posada, 2006). We set four Metropolis-coupled Markov chain Monte Carlo chains with 10,000,000 generations to get the best convergence of the chain with a sampling sequence of 1000 generations. The average standard deviation of split frequencies was used to assess the convergence of the two runs. We discarded the first 20% as burn-in, and constructed a 50%-majority rule consensus tree with the remaining trees. We also constructed networks of haplotypes and ribotypes with NETWORK 4.2.0.1 (Bandelt et al., 1999) to detect the network connection of haplotypes.

Divergence time between lineages were inferred by BEAST software (Drummond and Rambaut, 2007). Before the BEAST run, we tested if the strict clock model was suitable using a likelihood ratio test in PAUP 4.0b10. As the results showed that molecular clock could not be rejected (2logeLR = 2.76, df = 18, *P* > 0.05) for the ITS data set, we set the inference parameter as GTR + G model and a strict molecular clock, a constant population size coalescent tree prior and a UPGMA starting tree. We set 20,000,000 MCMC generations with sampling frequency as every 2,000 generations, and the first 5,000,000 generations as burn-in. Tracer (Drummond and Rambaut, 2007) was used to examine convergence of chains. We did not find reliable fossils that could be used for dating analysis of *R. fastigiata*, so we conducted dating based on substitution rate for ITS in shrubs and herbs reported by previous study (6.075 ± 1.590 × 10^-9^ s/s/y; Richardson et al., 2001).

### Population Genetics

Molecular diversity index including haplotype diversity (*h*) and nucleotide diversity (π) at both population and species level were calculated with DnaSP v5 (Librado and Rozas, 2009). We used PERMUT^1^ to estimate average gene diversity with a population (*H*_S_), total gene diversity (*H*_T_) and two population divergence index (*G*_ST_, *N*_ST_). We also performed 1,000 permutations replication to test the significant level. If *N*_ST_ value is significantly higher than *G*_ST_, more close related haplotypes will cluster (Zhang et al., 2005), indicating the existence of phylogeographic structure. To infer if geographic distance is correlated with genetic distance (isolation by distance), we conducted a Mental test on matrices of pair-wise geographic distance and *F*_ST_ using ARLEQUIN v 3.5 (Excoffier and Lischer, 2010) with 1,000 random permutations.

We performed a spatial analysis of molecular variance (SAMOVA) with SAMOVA v 1.0 (Dupanloup et al., 2002). This program finds the best *K*-value (number of geographic groups) by maximizing *F*_CT_ value between *K* groups of geographically adjacent populations. In our study, we set *K* from 2 to 10. After defining geographic groups, the amount of variation among populations within a group and a population were calculated by the hierarchical analysis of molecular variance (AMOVA) in ARLEQUIN v 3.5 (Excoffier and Lischer, 2010). A non-parametric permutation procedure with 1,000 permutations was conducted to test significant difference. Average *F*_ST_ between revealed geographic groups were also calculated with the same software.

To identify signatures of demographic expansion of populations and clades on the plastid tree, we estimated Tajima’s *D* and Fu’s *F*s values (Tajima, 1989; Fu, 1997). Significantly negative *D* and *F*_S_ values are expected if the focal population experienced expansion, because excessive rare and new mutation will appear in an expansion scenario. We also conducted a mismatch distribution analysis (Rogers and Harpending, 1992; Schneider and Excoffier, 1999) to detect the population expansion scenario of *R. fastigiata*. We pooled the whole haplotypes of each clade because evidence showed that population structure had little effect on mismatching distribution (Rogers, 1995). The fitting degree of observed mismatch distributions to the expected distribution under a recent expansion model (Rogers and Harpending, 1992; Excoffier et al., 2005) were tested using 1,000 parametric bootstrap replicates with sum of squared deviations (*SSD*) and the raggedness index (*HRag*) of Harpending (1994). If one group has the signal of expansion, we used the parameter-value (τ) to estimate when this expansion happened with equation *t* = τ/2*u* (Rogers and Harpending, 1992; Rogers, 1995). Here *u* = μ × *k* × *g*, where μ is the substitution rate (s/s/y), *k* is the average length of sequence data used, and *g* is the generation time (*y*). In this study, *k* was 1,593 bp, and the substitution rate was set to 2 × 10^-9^ s/s/y (Yamane et al., 2006). We assumed 10 years for generation time according to previous study (Zhang et al., 2014).

We also performed an Extended Bayesian Skyline Plot (EBSP) analysis using BEAST2 (Bouckaert et al., 2014) to estimate the demographic change of *R. fastigiata*. Effective population size through time were estimated based on coalescent process. As our test did not reject the molecular clock hypothesis, we used a strict clock model. We run the four MCMC chains for 50,000,000 generations with a sampling frequency of every 5,000 generations. After visualizing in Tracer (Rambaut et al., 2018), we drew the plot using a custom script in R software (R Core Team, 2017).

### Ecological Niche Modeling

To better understand the potential range shift of *R. fastigiata*, we employed ENMs to model distribution change in response to glacial climatic oscillations. By combining our own data and online herbarium records (e.g., Chinese Virtual Herbarium, and Global Biodiversity Information Facility), we obtained a total of 149 spatially unique localities for the modeling. Data from online databases were checked by the author to exclude misidentification. MAXENT 3.3.3e (Phillips and Dudík, 2008) was used to model potential distribution area of *R. fastigiata* of two time points: the LGM and the present. We downloaded environmental layers of 19 bioclimatic variables (**Supplementary Table S4**) for the Last Glacial Maximum (LGM) and the current time from the WorldClim website at a spatial resolution of 2.5 arc-minutes (Hijmans et al., 2005). After excluding highly correlated climate variables by examining pairwise correlations, seven variables (**Supplementary Table S4**) with pairwise Pearson correlation coefficients below 0.7 were used. As other study stated, areas under the “receiver operating characteristic (ROC) curve” (AUC) (Peterson et al., 2008; Elith and Leathwick, 2009) values were used to evaluate the accuracy of each model prediction. The threshold for good performance was set to 0.7 (Fielding and Bell, 1997). Finally, we implemented DIVA-GIS 7.5 (Hijmans et al., 2001) to draw the suitable distributions ranges.

## Results

### Haplotype Variation and Distribution

**FIGURE 1:**
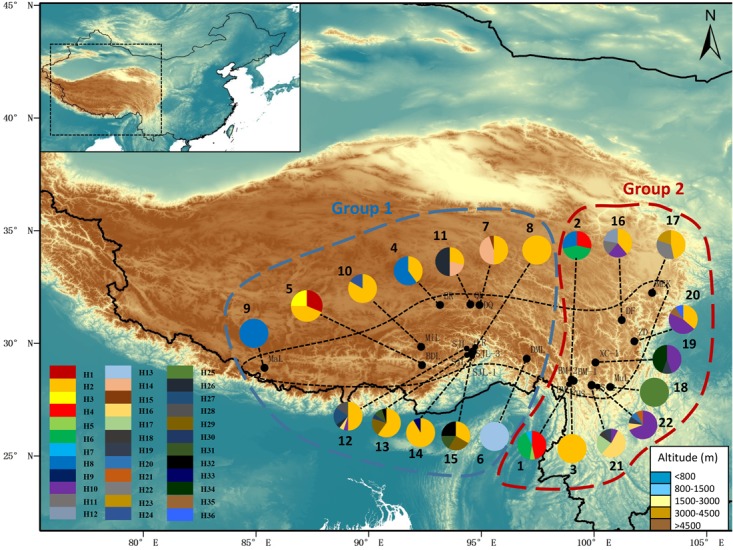
Map showing the sampling localities and the geographic distribution of haplotypes of *Rhodiola fastigiata* based on the cpDNA dataset. Pie charts show the proportion of haplotypes within each population. The numbers besides the circles represent population number as in **Supplementary Table S1**. Dashed line on the map indicates the distribution area of *R. fastigiata*. The two geographic groups defined by the SAMOVA analysis are also indicated by colored dashed lines.

After alignment, the total length of the combined cpDNA sequence was 1,593 bp. Thirty-six haplotypes based on 36 nucleotide substitutions were detected (including one outgroup individual; **Supplementary Table S2**). Unique sequences from each fragment were deposited in GenBank with accession number MH023238-MH023277. Haplotype diversity at the species level was *h* = 0.852, varying in different populations from 0.000 to 0.803 (SJL-4) (**Supplementary Table S1**). At the species level, nucleotide diversity was π = 0.0029, with a range from 0.000 to 0.0036 in different populations (**Supplementary Table S1**). Within-population gene diversity (*H*_S_) was significantly lower than total gene diversity (*H*_T_) (0.494 and 0.877, respectively, *P* < 0.05; **Table 1**). Twenty-three of the 36 haplotypes were found in only one population (**Supplementary Table S2**), and others occurred in at least two or more populations with H2 found in 13 of the 22 sampled populations (**Figure 1**). In the 22 populations, 5 only harbored a single haplotype, while SJL-1 harbored 6 haplotypes (**Figure 1**).

**Table 1:** Genetic diversity and genetic differentiation of 22 populations of *Rhodiola fastigiata* at the species level and group levels.

Plastid DNA					ITS				
		
Groups	*H*_S_	*H*_T_	*π* (×10^-3^)	*G*_ST_	*N*_ST_	*H*_S_	*H*_T_	*π* (×10^-3^)	*G*_ST_	*N*_ST_
Total	0.494 (0.0623)	0.877 (0.0387)	2.89	0.437 (0.0742)	0.621 (0.0743)^∗∗^	0.216 (0.0673)	0.746 (0.0705)	1.80	0.711 (0.0838)	0.719 (0.0907)^ns^
Group 1	0.482 (0.0772)	0.777 (0.0581)	1.87	0.380 (0.1071)	0.405 (0.0786)	0.358 (0.1370)	0.676 (0.1327)	1.20	0.470 (0.1786)	0.405 (0.1562)
Group 2	0.629 (0.0464)	0.907 (0.0438)	2.36	0.306 (0.0228)	0.228 (0.0832)	0.145 (0.0638)	0.155 (0.0638)	0.45	0.065 (NC)	0.104 (NC)^∗^
Group 3	0.000 (0.0000)	0.000 (0.0000)	0.00	–	–	0.000 (0.0000)	0.000 (0.0000)	0.00	–	–

*^∗^NST is significantly different from GST (P < 0.05); ^∗∗^NST is significantly different from GST (P < 0.01); ns, not significant.*

### Ribotype Variation and Distribution

**FIGURE 2:**
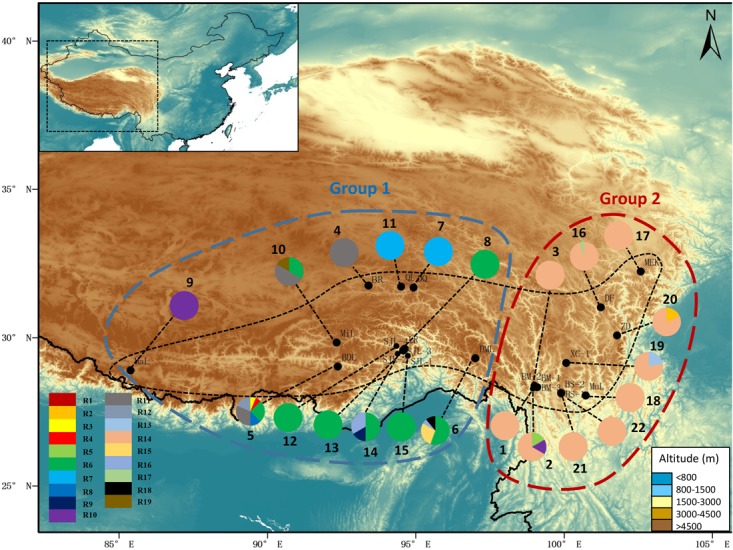
Map showing the sites of sampled populations and the geographic distribution of ribotypes of *R. fastigiata* based on the ITS dataset. Pie charts show the proportion of ribotypes within each population. The numbers besides the circles represent population number as in **Supplementary Table S1**. Dashed line on the map indicates the distribution area of *R. fastigiata*. The two geographic groups defined by the SAMOVA analysis are also indicated by colored dashed lines.

The aligned ITS sequences was 640 bp in length. We detected 19 ribotypes, defined by 18 nucleotide substitutions (including one outgroup individual; **Supplementary Table S3**). Sequences were uploaded to GenBank with accession number MH 023219-MH023237. At the species level, ribotype diversity was 0.703, ranging from 0.000 to 0.833 (**Supplementary Table S1**). Nucleotide diversity π = 0.0018 at the species level, varying from 0.000 to 0.0027 in different populations. Population BDL had the highest ribotype diversity, while BM-2 had the highest π value. Similar to the plastid dataset, within-population gene diversity (*H*_S_) was significantly lower than total gene diversity (*H*_T_) (0.216 and 0.746, respectively; **Table 1**). Of the 19 ribotypes, 13 only occurred in one population (**Supplementary Table S3**). 14 out of the 22 populations only harbored one ribotypes, with population BDL had 6 ribotypes, the most in all populations (**Figure 2**).

### Population Structure

The permutation tests of plastid data demonstrated that *N*_ST_ was significantly higher than *G*_ST_ (**Table 1**), indicating presence of phylogeographic structure. However, we failed to detect a significant phylogeographic structure from the ITS data. In the SAMOVA analysis for both cpDNA and ITS datasets *F*_CT_ value reached a plateau when *K* = 3 (**Supplementary Figure S1**). In the plastid dataset, the first group consisted of 11 populations on the QTP plateau, and group two comprised 10 populations in the Hengduan Mountains. Group three only had one population on the plateau and was thus merged to group 1 in the discussion hereafter (**Figure 1**). For the ITS data, we also detected three geographic groups, two of them corresponding to the QTP plateau and the Hengduan Mountains, respectively, and the third group with only two ribotypes was merged to group 1 hereafter as they were also on the QTP plateau. Average *F*_ST_ between revealed geographic groups are shown in **Table 2**. AMOVA analysis showed that for the cpDNA dataset, 31.89% of the total variations occurred among groups decided by SAMOVA, and 44.77% of the total variations were with populations. For the ITS dataset, variations among groups also accounted for 71.26% of the total variation (**Table 3**). The Mantel test based on both datasets showed a significant correlation between genetic distance and geographic distance: a pattern of isolation-by-distance (cpDNA: *r* = 0.387, *P* < 0.05; ITS: *r* = 0.423, *P* < 0.01).

**Table 2:** Pairwise comparisons of *F*_ST_ among regions estimated from internal transcribed spacer (ITS) sequences (upper part) and cpDNA sequences (lower part) of *Rhodiola fastigiata*.

	Group 1	Group 2	Group 3
Group 1		0.7492	0.6518
Group 2	0.5368		0.9432
Group 3	0.6321	0.43610	

*All values are significant at the 0.01 level in a permutation tests (1000 permutations).*

**Table 3:** Analysis of molecular variance (AMOVA) of cpDNA haplotypes and ITS ribotypes for *Rhodiola fastigiata* populations.

ITS					pDNA				
		
Source of variation	df	SS	VC	PV(%)	*F*-statistics	df	SS	VC	PV(%)	*F*-statistics
Among groups	2	247.95	1.64	71.26	*F*_SC_ = 0.25176^∗^	2	463.92	3.59	31.89	*F*_SC_ = 0.34274^∗^
Among populations	19	42.938	0.17	7.24	*F*_ST_ = 0.78494^∗^	19	748.28	2.63	23.35	*F*_ST_ = 0.55231^∗^
Within populations	228	112.97	0.50	21.51	*F*_CT_ = 0.71258^∗^	265	1336.86	5.04	44.77	*F*_CT_ = 0.31886^∗^
Total	248	403.851	2.30	–	–	286	2549.05	11.27	–	–

*df, degrees of freedom; SS, sum of squares; VC, variance components; PV, percentage of variation. *F*_*SC*_, correlation within populations relative to group; *F*_*ST*_, correlation within populations relative to total; *F*_*CT*_, correlation within groups relative to total. ^∗^*P* < 0.001, 1000 permutations*.

### Phylogenetic Relationships and Lineages Divergence

**FIGURE 3:**
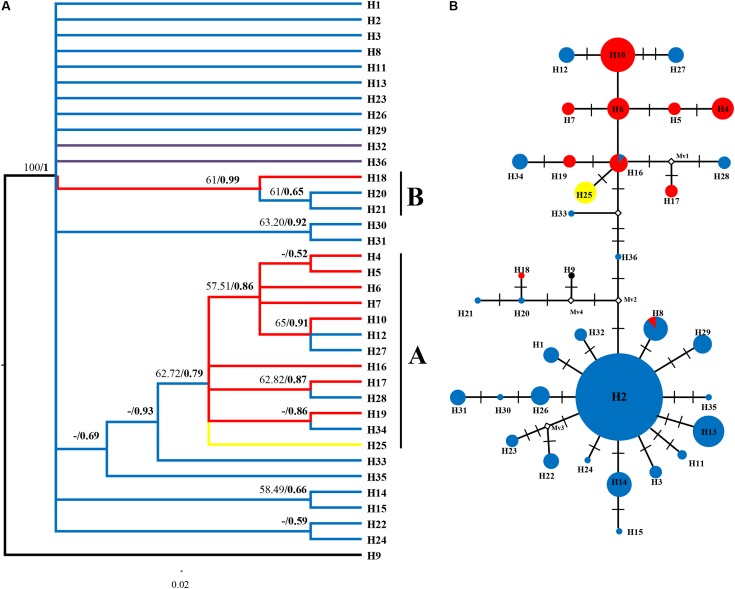
Phylogenetic relationships based on cpDNA haplotypes. **(A)** The Bayesian tree topology of the 36 cpDNA haplotypes detected in *R. fastigiata*. Numbers above the branches are MP bootstrap support values (left) and Bayesian posterior probability (right). Different branch color represents geographic groups defined by the SAMOVA analysis. H9 represents outgroup sequence of *R. coccinea*. **(B)** NETWORK-derived genealogic relationships. The sizes of the circles in the network were proportional to the observed frequencies of the haplotypes in each population. The white diamonds represent missing chlorotypes. Different color of circles represents geographic groups defined by the SAMOVA analysis.

**FIGURE 4:**
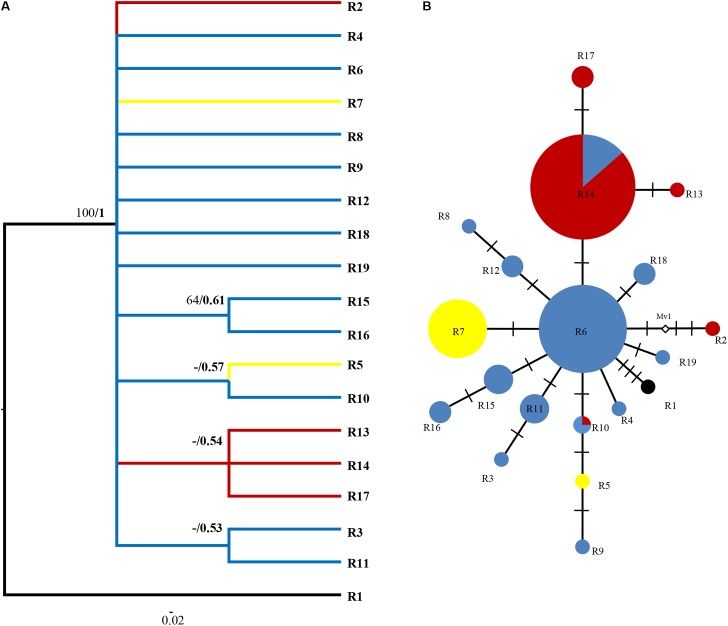
Phylogenetic relationships based on ITS ribotypes. **(A)** The Bayesian tree of the 19 ITS ribotypes detected in *R. fastigiata*. Numbers above the branches are MP bootstrap support values (left) and Bayesian posterior probability (right). R1 represents outgroup sequence of *R. coccinea*. **(B)** NETWORK-derived genealogic relationships. The size of the circles in the network is proportional to the observed frequencies of the haplotypes in each population. The white diamonds represent missing ribotypes. Different color of circles represents geographic groups defined by the SAMOVA analysis.

We only showed the Bayesian tree as MP and Bayesian methods yield largely congruent topology for both datasets (**Figures 3A**, **4A**). In the plastid data tree, all haplotypes of *R. fastigiata* formed a well-supported clade (**Figure 3A**). Two clades (Clades A and B) could be found. Clade A comprised 13 haplotypes and clade B only 3. The haplotype network (**Figure 3B**) depicted relationships between haplotypes more clearly: all haplotypes can be divided into two groups, corresponding to the two geographic groups determined by SAMOVA analysis (Although our SAMOVA analysis revealed a *K*-value of 3, group 3 only had one haplotype).

**FIGURE 5:**
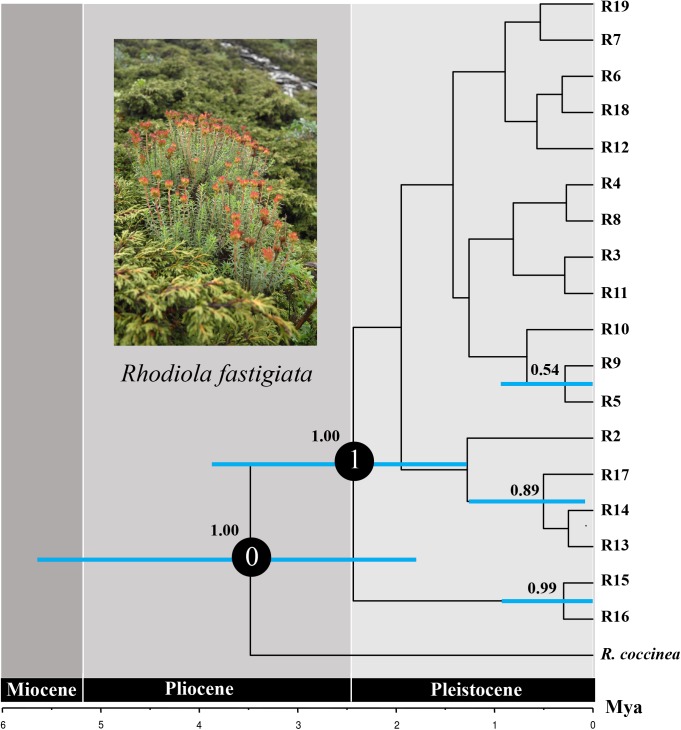
Divergence time of *R. fastigiata* based on ITS ribotypes estimated with BEAST. Blue bars indicate 95% highest posterior density intervals. Numbers on the branch represent posterior probability of each node. The vertical red line on the tree tips indicates the Holocene.

For the ITS data, ribotypes were also divided into two geographic groups (indicated by different color in **Figure 4**). Similar to the plastid data, although our SAMOVA analysis revealed three geographic groups, group 3 only comprised two ribotypes that had the same distribution area with group 1. Dating analysis based on the ITS data showed that *R. fastigiata* diverged from its closest relatives at 3.47 Mya (Node 0; 95% HPD: 1.82–5.51 Mya) in the Pliocene (**Figure 5**). Further divergence occurred after 2.41 May (Node 1; 95% HPD: 1.20–3.72 Mya), in the Pleistocene.

### Demographic Analyses

We did not detect any signal of population expansion of *R. fastigiata* at the species level or for any clades of the tree: under a population expansion model, all haplotypes as a whole showed a multi modal mismatch distribution in the mismatch distribution analysis (**Supplementary Figure S2**). Furthermore, *SSD* and the raggedness index significantly differed from expected values if an expansion hypothesis is true (**Table 4**). No significantly negative value of Tajima’s *D* and Fu’s *F*s were also revealed (**Table 4**). Our Extended Bayesian Skyline Plot (EBSP) analysis revealed a similar pattern: the effective population size of *R. fastigiata* remained stable in last 0.82 million years, with a slight increasing toward the present time (**Supplementary Figure S3**).

**Table 4:** Results of the mismatch distribution analysis and neutrality tests of the three multiple-haplotype pDNA clades.

Haplotype group	τ	*t* (Mya)	*SSD*	*P*-value	Raggedness index	*P*-value	Taijima’s *D*	*P*-value	*F*_S_	*P*-value
Clade A	2.340	–	0.042	0.914	0.222	0.987	–	0.941	-0.693	0.053
Clade B	44.18	–	0.026	0.36	0.056	0.16	0.0552	0.584	22.74	0.998
Total	50.061	–	0.028	0.047	0.044	0.013	-0.850	0.211	6.555	0.860

### Species Distribution Models for *R. fastigiata*

**FIGURE 6:**
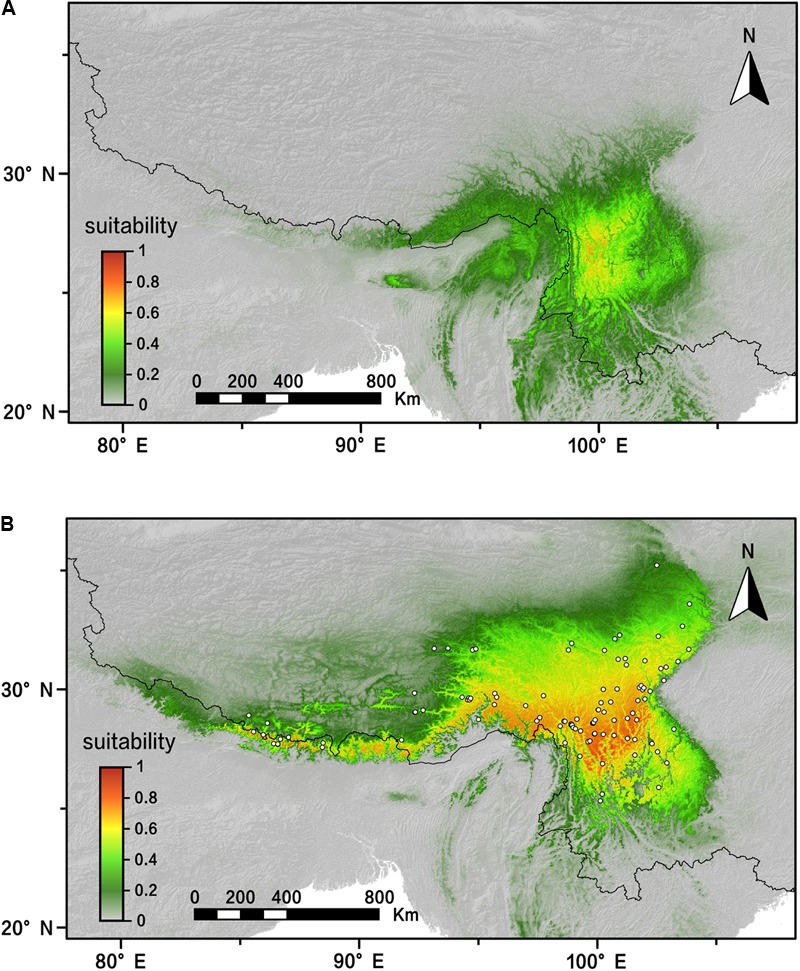
Distribution dynamics of *R. fastigiata* during the present day and the LGM based on species distribution modeling using MAXENT. Predicted distributions are shown for **(A)** the LGM model, **(B)** present. White squares represent presence locations (field survey data). LGM, last glacial maximum.

The AUC value (AUC > 0.98; standard deviation [SD] < 0.01) showed that our modeling was effectively predictive according to 20 MAXENT replicating runs. The predicted distribution of *R. fastigiata* of present day is generally the same as the actual distribution today (**Figure 6B**). Our result also showed that the distribution of *R. fastigiata* during the LGM was slightly smaller than present distribution, especially in the eastern QTP and northern part of the Hengduan Mountains (**Figure 6A**). This pattern is in accordance with the EBSP results shown in the last section.

## Discussion

### Phylogeographic History of *R. fastigiata*

*Rhodiola fastigiata* inhabits the subnival belt a little below the permanent snowline, which represents the highest terrestrial habitat that an organism can occupy (Körner, 2003). As species growing in these habitats are cold adapted, we hypothesized that these species would expand their distribution area as temperature went down in the glacial times, and vice versa. Repeated expansion and contraction of distribution area will create an island-like distribution pattern. Our data was consistent with this hypothesis. In the AMOVA analysis, both markers showed a high *F*_ST_ value (ITS, 0.78; cpDNA, 0.55), indicating great difference among populations, and most variation was at group and population level (**Table 3**). In addition, *N*_ST_ was significantly greater than *G*_ST_ for the cpDNA data (**Table 1**), indicating significant phylogeographic structure. However, such phylogeographic structure was not detected in the ITS data. This results indicated that gene flow via pollen is more efficient than via seeds in *R. fastigiata*, which is congruent with its congeneric species *R. kirilowii* (Zhang et al., 2014). Another reason for lack of significant phylogeographic structure in the ITS data is that 63.6% of the sampled population harbored only one haplotype (**Figure 2**).

In the spatial analysis of molecular variance (SAMOVA), we detected two groups in both markers. The two groups largely corresponded to the Hengduan Mountains area and the QTP plateau platform. A high genetic differentiation among groups were detected by the AMOVA analysis (**Table 3**), and confirmed by our pairwise *F*_ST_ calculation among three groups (**Table 2**). Molecular dating analysis showed that these two groups diverged at 1.23 Mya (95% HPD: 0.56–2.01 Mya) based on the ITS dataset. Thus we can infer that the Pleistocene climatic oscillations might have triggered the divergence of the two groups. Populations of *R. fastigiata* might have retreated to isolated glacial refugia in the HM and QTP area during the glacial period. We detected no range expansion both at the species level and in each of the clades of the cpDNA tree (**Figure 3** and **Table 4**). Ecological niche modeling results also support the scenario that later glacial time has little to do with the distribution of the studies species (**Figure 6**). This result is in congruence with two recent studies focused on several subnival species in the same area (Luo et al., 2016, 2017), in which ENM demonstrated that those species’ distribution area remained stable in the Quaternary glaciations.

### Comparison to Other Species of *Rhodiola*

Gao et al. (2012) studied the Quaternary history of *R. alsia*, which is the first phylogeographic study in *Rhodiola*. *R. fastigiata* and *R. alsia* are very similar in morphology except tiny difference in leaf shape and length of carpel. Their work with two cpDNA markers (*rpl*20-*rps*12 and *trn*S-G) and ITS markers showed that there were probably three potential refugia, one in the Hengduan Mountain area and the other two on the QTP platform (Gao et al., 2009, 2012). The dated divergence time of three clades was 0.35–0.87 Mya ago, during the Pleistocene. The phylogeographic pattern of *R. alsia* is very similar to *R. fastigiata* with regard to refugia’s location. We also revealed a divergence between the QTP plateau group and the Hengduan Mountains group, indicating that refugia existed in both regions. However, the divergence of the two groups was 1.23 Mya (95% HPD: 0.56–2.01 Mya). Although there is overlap between the 95% HPDs, the intra-specific divergence time of *R. fastigiata* is slightly earlier than that of *R. alsia*. Besides, Gao et al. (2009) used mismatch distribution analysis and showed that population expansion had occurred in the history of *R. alsia*, whereas such pattern was not revealed for *R. fastigiata* in current study. A relatively stable population demography was also demonstrated in our ENM data (**Figure 6**) and EBSP analysis (**Supplementary Figure S3**). A recent study also showed that the Quaternary glacial had had little impact on four subnival plants on the QTP (Luo et al., 2016). Study of another morphological similar species, *R. crenulata*, which also inhabits subnival areas of the QTP, showed that there had been two glacial refugia, i.e., the HM and QTP platform (Zhang et al., Unpublished). However, mismatch distribution analysis showed that a recent population expansion happened in ca. 0.31 Mya. This expansion was also detected in the EBSP analysis.

Even though *R. fastigiata* and *R. alsia* have similar physiological and life history traits, they had different demographic histories in the Quaternary glacial periods, at least based on our analyses. Although previous literature has demonstrated that glacial histories of species with different features (e.g., cold tolerance, drought tolerance, life cycle, and dispersal ability) were also distinct (Shafer et al., 2010; Stewart et al., 2010), our results showed that even species with similar physiology traits in the same genus could have different response to glacial climatic oscillations. This indicates that factors other than climate would affect population histories of plants. One hypothesis is that the changing balance of coevolved interactions between hosts and their specialized pathogens could drive population dynamics (Ricklefs, 2015). Further studies are needed to test this hypothesis. However, this conclusion needs to be treated with caveats, as it should be tested using statistical phylogeography or spatially explicit phylogeographic analyses with balanced sampling and molecular markers (Lemmon and Lemmon, 2008; Barrow et al., 2015).

Refugia on the QTP was also identified for *R. dumulosa* (Hou and Lou, 2014), a cold adapted species with elongated rhizomes and persistent flowering stems. These traits may confer it the ability to survive in the refugia of the QTP platform. On the contrary, *R. kirilowii*, a species with no persistent flower stems, only occupied the Hengduan Mountain areas as major refugia. Refugia were also detected in other area of species’ distribution in Tianshan area in Xinjiang. Interestingly, a recent study of *R. chrysanthemifolia*, a woodland- and shrubbery-inhabited herbaceous species, used two cpDNA markers and ITS data and uncovered that there had been multiple microrefugia during the LGM, even earlier glaciations, a pattern also found in two sympatric tree and shrub species (Gao et al., 2016). These wood species might have provided it suitable habitats for surviving in the glacial periods. This pattern was further supported by a recent study including more species and populations (Li et al., 2018). Although the distribution area of *R*. *chrysanthemifolia* is similar to that of *R. crenulata*, *R. fastigiata* and *R. alsia*, their mirco-habitats are distinct: *R. chrysanthemifolia* is lower in elevation and mainly grows on the forest floor and in alpine shrubberies. This example showed that micro-inhabits play an important role in how plants respond to glacial climatic oscillations.

It is interesting to compare genetic diversity of hermaphrodite species and dioecious species, because Diocy is thought to evolve to promote outcrossing and increase heterozygosity (Barrett, 2002). We compared cpDNA and ITS diversity between hermaphrodite species and dioecious species, and no significant difference for both datasets was detected (ITS: *p* = 0.23; cpDNA: *p* = 0.36). Although this result needs to be treated with caution because of different sampling strategy in different studies, it provides evidence that dioecy in *Rhodiola* was not evolved to promote cross pollination. Therefore, the alternative hypothesis proposed by Darwin (1877) that plants evolve dioecy to allocate reproductive energy might be right. This makes sense because species like *Rhodiola* often grow on very high altitude and harsh environment where the growing season is very short.

## Author Contributions

J-QZ conceived the ideas and wrote the manuscript. D-LZ, R-WZ, and W-YS conducted the experiments and collected the data. W-JS and D-LZ analyzed the data.

## Conflict of Interest Statement

The authors declare that the research was conducted in the absence of any commercial or financial relationships that could be construed as a potential conflict of interest.

## Footnotes

1 http://www.pierroton.inra.fr/genetics/labo/Software/Permut

## Supplementary Material

FIGURE S1 Correlation between the *F* statistics and grouping number (*K* = 2–10) from the SAMOVA results. **(a)** cpDNA; **(b)** ITS.

FIGURE S2 Historical demography for overall populations and in each regional group based on the plastid DNA dataset. Clades A and B corresponded to the Bayesian phylogenetic tree in **Figure 3**. Mismatch distribution showing histogram of observed mismatch frequencies and best-fit curve of the sudden expansion model.

FIGURE S3 Extended Bayesian Skyline Plot (EBSP) results calculated by BEAST2. Grey shade indicates 95% CPD of the effective population size.

TABLE S1 Locations of populations of Rhodiola sect. Trifida sampled, sample sizes (N), frequencies of cpDNA haplotypes and ITS sequences per population, and estimates of haplotype diversity and nucleotide diversity for chlorotypes and ribotypes within populations.

TABLE S2 Haplotype composition of 22 sampled based on cpDNA dataset for populations.

TABLE S3 Ribotype composition of 21 sampled based on ITS dataset for populations.

TABLE S4 Bioclimatic variables (named BIO1 to BIO19) from WorldClim (Hijmans et al., 2005). Variables marked with an asterisk (^∗^) were used for the climatic niche models of *Rhodiola fastigiata*.
