# Supplementary figures and images for: Climate Is Not All: Evidence From Phylogeography of Rhodiola fastigiata (Crassulaceae) and Comparison to Its Closest Relatives
Source: Front Plant Sci. 2018 Apr 10;9:462. doi: 10.3389/fpls.2018.00462 (PMC5912201; doi:10.3389/fpls.2018.00462)

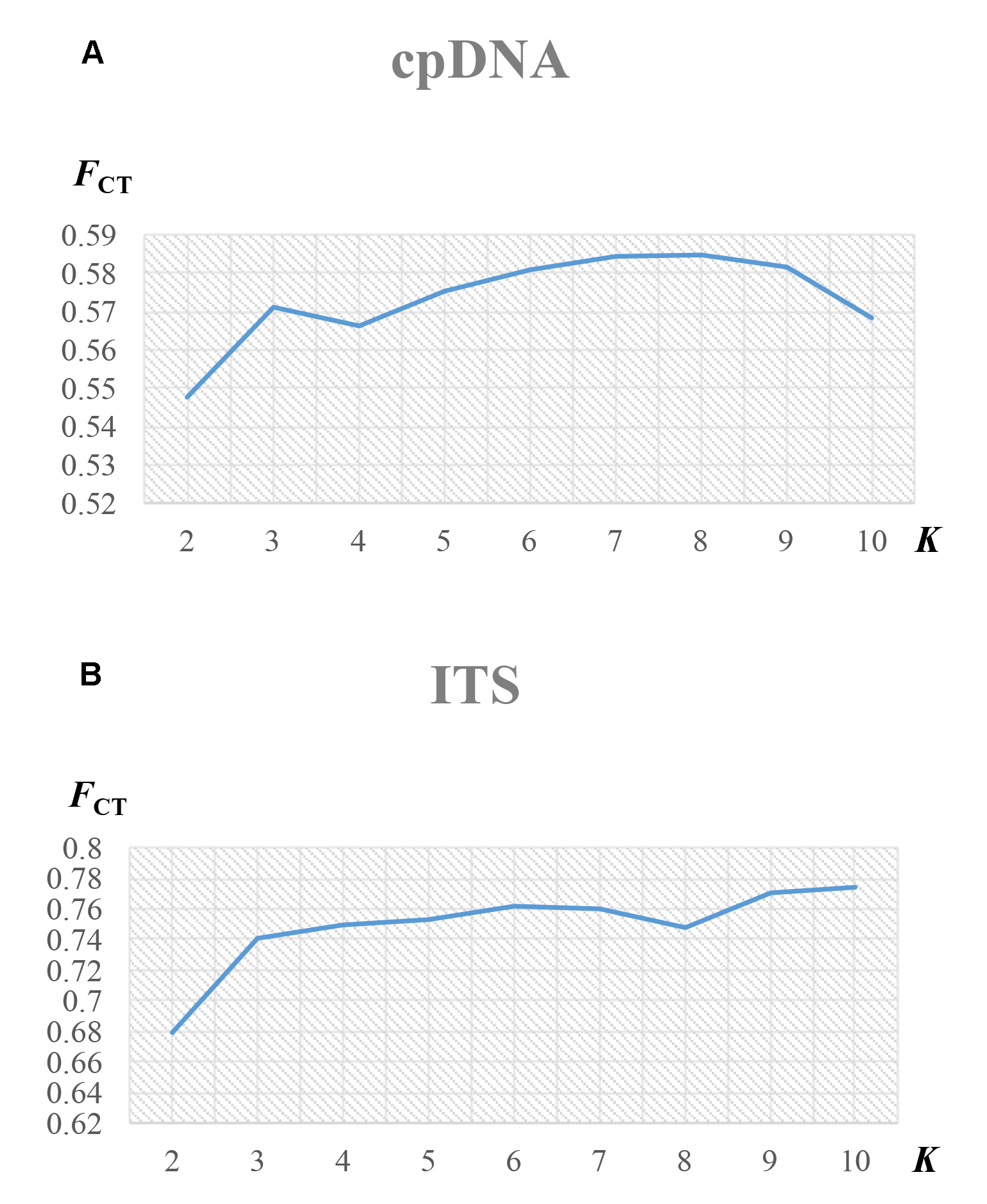

Supplement: FIGURE S1 — Correlation between the F statistics and grouping number (K = 2–10) from the SAMOVA results. (a) cpDNA; (b) ITS. [file Image_1.TIF]

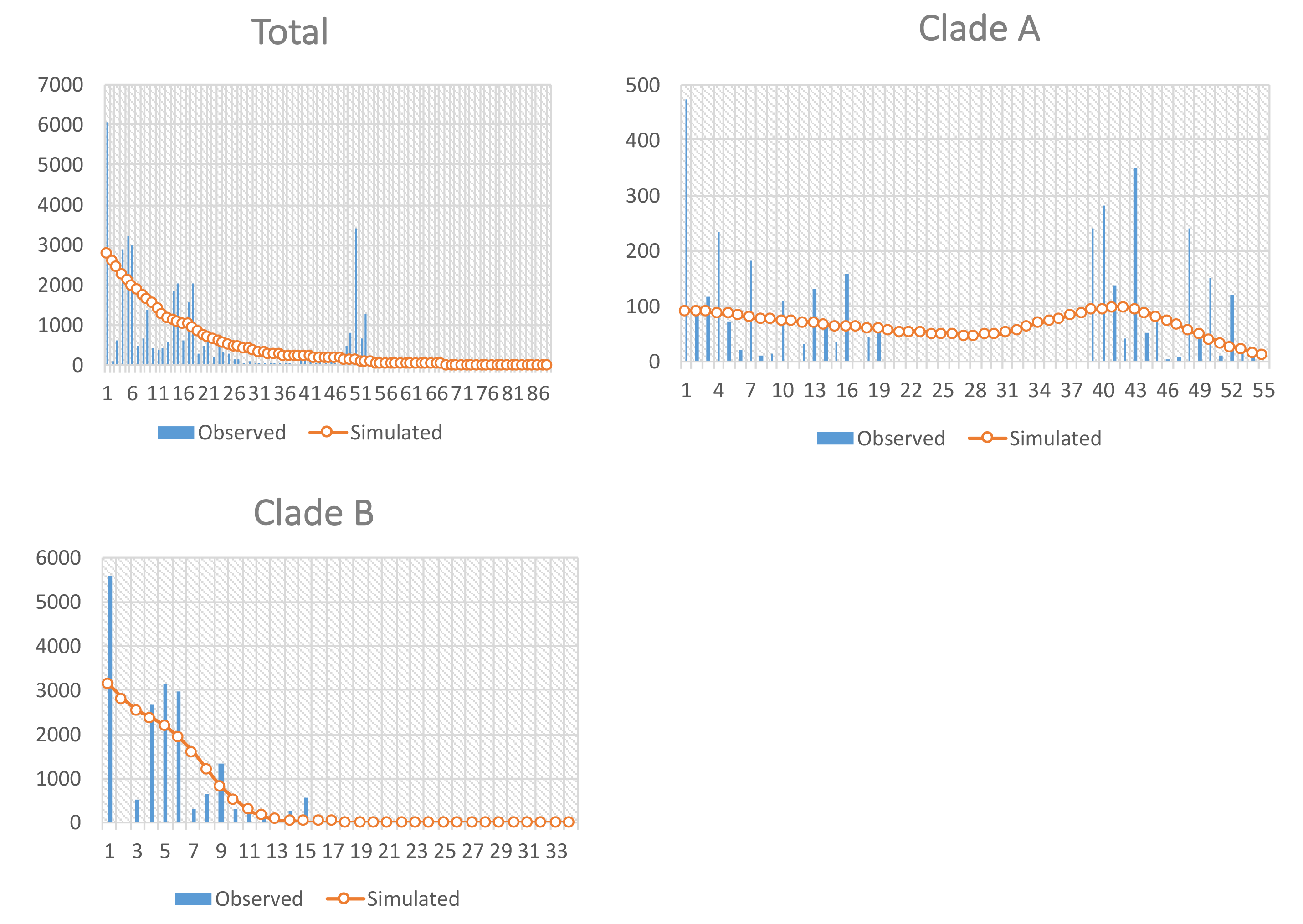

Supplement: FIGURE S2 — Historical demography for overall populations and in each regional group based on the plastid DNA dataset. Clades A and B corresponded to the Bayesian phylogenetic tree in Figure 3. Mismatch distribution showing histogram of observed mismatch frequencies and best-fit curve of the sudden expansion model. [file Image_2.TIF]

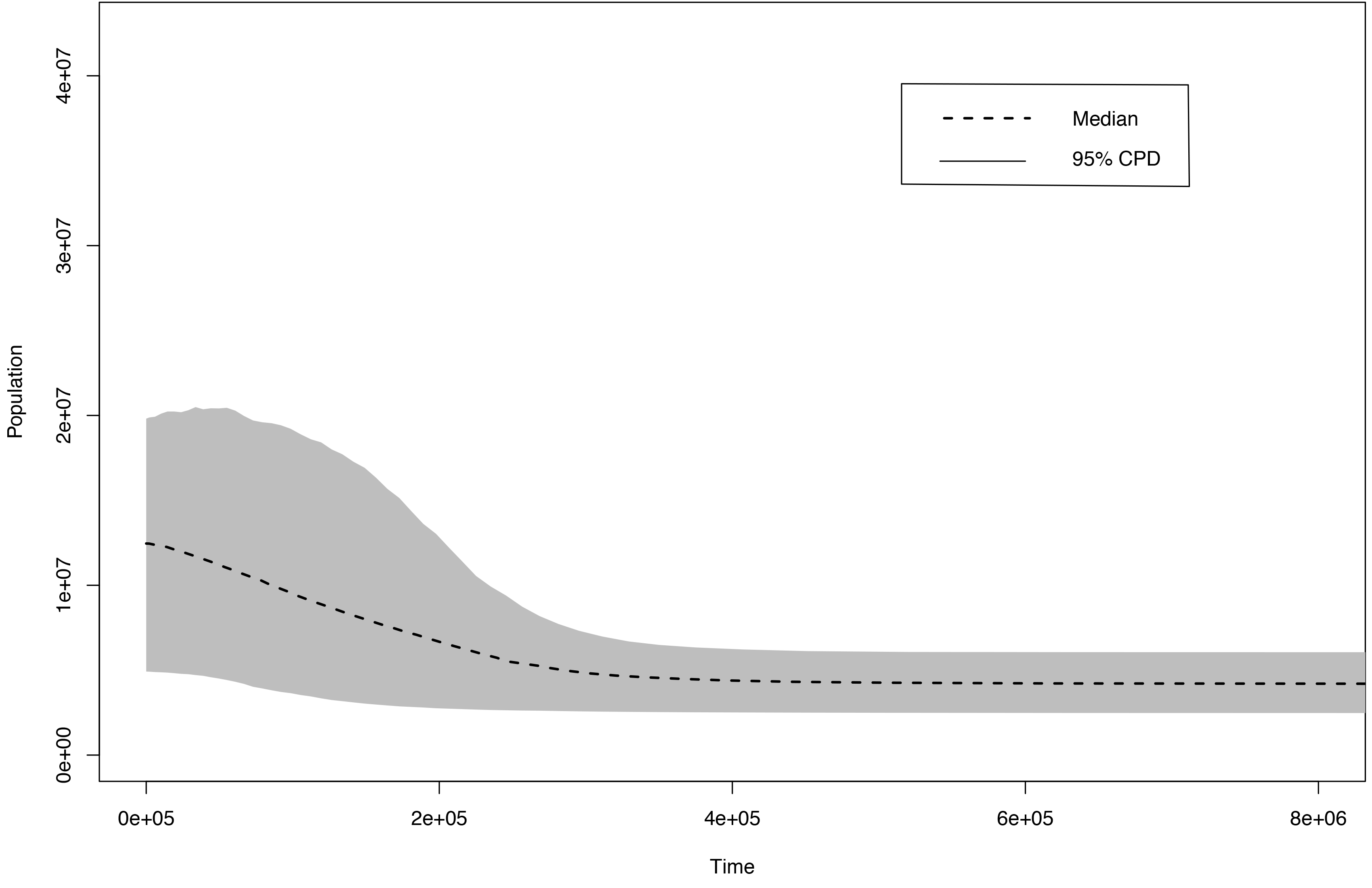

Supplement: FIGURE S3 — Extended Bayesian Skyline Plot (EBSP) results calculated by BEAST2. Grey shade indicates 95% CPD of the effective population size. [file Image_3.TIF]
