# Supplementary material for: Climate Is Not All: Evidence From Phylogeography of Rhodiola fastigiata (Crassulaceae) and Comparison to Its Closest Relatives
Source: Front Plant Sci. 2018 Apr 10;9:462. doi: 10.3389/fpls.2018.00462 (PMC5912201; doi:10.3389/fpls.2018.00462)
Supplement: TABLE S1 — Locations of populations of Rhodiola sect. Trifida sampled, sample sizes (N), frequencies of cpDNA haplotypes and ITS sequences per population, and estimates of haplotype diversity and nucleotide diversity for chlorotypes and ribotypes within populations. [file Table_1.DOC]

**Table S1.** Locations of populations of *Rhodiola* sect. *Trifida* sampled, sample sizes (N), frequencies of cpDNA haplotypes and ITS sequences per population, and estimates of haplotype diversity and nucleotide diversity for chlorotypes and ribotypes within populations

| Chlorotypes | | | | | | | | | | Ribotypes | | |
| --- | --- | --- | --- | --- | --- | --- | --- | --- | --- | --- | --- | --- |
| Population | |  | Lat. (N) | Long. (E) | Alt. (m) | N | Haplotypes nos. | *h* (SD) | *π* (SD) in ‰ | ITS sequences nos. | *h* (SD) | *π* (SD) in ‰ |
| 1 | BM-1 | Baima Mt. | 28º20'02" | 99º05'59" | 4312 | 17 | H4(8)H5(1)H6(7)H7(1) | 0.64(0073) | 0.0007(0.0003) | H14(17) | 0 | 0 |
| 2 | BM-2 | Baima Mt. | 28º23'06" | 99º00'34" | 4312 | 7 | H4(2)H6(3)H8(2) | 0.762(0.115) | 0.0026(0.0012) | H5(1)H10(1)H14(5) | 0.524(0.209) | 0.0027(0.0016) |
| 3 | BM-3 | Baima Mt. | 28º20'02" | 99º05'59" | 4312 | 12 | H2(12) | 0 | 0 | H14(12) | 0 | 0 |
| 4 | BR | Biru | 31º42'14" | 93º10'17" | 4436 | 20 | H2(8)H8(12) | 0.505(0.056) | 0.0003(0.0002) |  |  |  |
| 5 | BDL | Budangla Mt. | 29º02'01" | 92º22'18" | 4613 | 16 | H1(5)H2(7)H3(4) | 0.692(0.058) | 0.0006(0.0003) | H3(1)H4(1)H6(4)H8(2)H11(5)H12(3) | 0.833(0.056) | 0.0022(0.0010) |
| 6 | DML | Demula Mt. | 29º18'36" | 97º00'59" | 4900 | 18 | H13(18) | 0 | 0 | H6(10)H15(5)H16(1)H18(2) | 0.771(0.063) | 0.0020(0.0011) |
| 7 | DQ | Dingqing | 31º41'34" | 94º55'31" | 4959 | 20 | H2(10)H14(9)H15(1) | 0.574(0.055) | 0.0004(0.0003) | H7(20) | 0 | 0 |
| 8 | LR | Lairui | 29º37'58" | 94º37'30" | 4350 | 1 | H2(1) | 0 | 0 | H6(1) | 0 | 0 |
| 9 | MaL | Mala Mt. | 28º54'14" | 85º22'64" | 5016.8 | 3 | H8(3) | 0 | 0 | H10(3) | 0 | 0 |
| 10 | MiL | Mila Mt. | 29º50'02" | 92º20'02" | 5225 | 6 | H2(5)H24(1) | 0.333(0.215) | 0.0002(0.0003) | H6(2)H11(3)H19(1) | 0.8(0.172) | 0.0023(0.0014) |
| 11 | QL | Qiala Mt. | 31º43'17" | 94º30'55" | 4339 | 14 | H2(4)H14(3)H26(7) | 0.670(0.082) | 0.0006(0.0003) | H7(14) | 0 | 0 |
| 12 | SJL-1 | Sejila Mt. | 29º33'62" | 94º34'58" | 4728 | 20 | H2(10)H10(1)H16(1)H26(1)H27(4)H28(3) | 0.716(0.087) | 0.0036(0.001) | H6(20) | 0 | 0 |
| 13 | SJL-2 | Sejila Mt. | 29º33'62" | 94º34'58" | 4728 | 20 | H2(12)H29(4)H30(1)H31(2)H32(1) | 0.616(0.106) | 0.0014(0.0007) | H6(20) | 0 | 0 |
| 14 | SJL-3 | Sejila Mt. | 29º33'62" | 94º34'58" | 4728 | 12 | H2(11)H33(1) | 0.167(0.134) | 0.0006(0.0006) | H6(6)H9(2)H16(4) | 0.810(0.130) | 0.0024(0.0016) |
| 15 | SJL-4 | Sejila Mt. | 29º33'62" | 94º34'42" | 4729 | 12 | H2(4)H29(3)H31(2)H32(3) | 0.803(0.063) | 0.0014(0.0007) | H6(12) | 0 | 0 |
| 16 | DF | Daofu | 31º01'28" | 101º14'20" | 4200 | 18 | H2(7)H10(4)H11(3)H12(4) | 0.765(0.055) | 0.0032(0.0009) | H17(2)H14(16) | 0.228(0.102) | 0.0003(0.0004) |
| 17 | MEK | Ma'erkang | 32º13'60" | 102º35'06" | 4300 | 15 | H2(7)H22(5)H23(3) | 0.676(0.070) | 0.0011(0.0005) | H14(15) | 0 | 0 |
| 18 | MuL | Muli | 28º03'26" | 100º43'40" | 4170 | 10 | H25(10) | 0 | 0 | H14(10) | 0 | 0 |
| 19 | XC-1 | Xiangcheng | 29º08'43" | 100º04'24" | 4640 | 9 | H10(4)H19(1)H34(4) | 0.75(0.112) | 0.0019(0.0007 | H13(2)H14(7) | 0.644(0.152) | 0.0021(0.0010) |
| 20 | ZD | Zheduo Mt. | 30º04'56" | 101º47'34" | 4256 | 11 | H2(4)H10(5)H35(1)H36(1) | 0.709(0.099) | 0.0030(0.0010) | H2(2)H14(9) | 0.4170.191 | 0.0021（0.0020） |
| 21 | HS-1 | Hong Mt. | 28º08'51" | 99º54'13" | 4494 | 13 | H10(1)H16(7)H17(3)H18(1)H19(1) | 0.692(0.119) | 0.0016(0.0012) | H14(13) | 0 | 0 |
| 22 | HS-2 | Hong Mt. | 28º07'09" | 99º54'04" | 4334 | 13 | H10(9)H16(1)H19(1)H20(1)H21(1) | 0.538(0.161) | 0.0023(0.0011) | H14(13) | 0 | 0 |
