# Supplementary material for: Climate Is Not All: Evidence From Phylogeography of Rhodiola fastigiata (Crassulaceae) and Comparison to Its Closest Relatives
Source: Front Plant Sci. 2018 Apr 10;9:462. doi: 10.3389/fpls.2018.00462 (PMC5912201; doi:10.3389/fpls.2018.00462)
Supplement: TABLE S3 — Ribotype composition of 21 sampled based on ITS dataset for populations. [file Table_3.DOC]

**Table S3**. Ribotype composition of 21 sampled based on ITS dataset for populations

| Population | 1 | 2 | 3 | 4 | 5 | 6 | 7 | 8 | 9 | 10 | 11 | 12 | 13 | 14 | 15 | 16 | 17 | 18 | 19 |
| --- | --- | --- | --- | --- | --- | --- | --- | --- | --- | --- | --- | --- | --- | --- | --- | --- | --- | --- | --- |
| BM-1 |  |  |  |  |  |  |  |  |  |  |  |  |  | 17 |  |  |  |  |  |
| BM-2 |  |  |  |  | 1 |  |  |  |  | 1 |  |  |  | 5 |  |  |  |  |  |
| BM-3 |  |  |  |  |  |  |  |  |  |  |  |  |  | 12 |  |  |  |  |  |
| BDL |  |  | 1 | 1 |  | 4 |  | 2 |  |  | 5 | 3 |  |  |  |  |  |  |  |
| DML |  |  |  |  |  | 10 |  |  |  |  |  |  |  |  | 5 | 1 |  | 2 |  |
| DQ |  |  |  |  |  |  | 20 |  |  |  |  |  |  |  |  |  |  |  |  |
| LR |  |  |  |  |  | 1 |  |  |  |  |  |  |  |  |  |  |  |  |  |
| MaL |  |  |  |  |  |  |  |  |  | 3 |  |  |  |  |  |  |  |  |  |
| MiL |  |  |  |  |  | 2 |  |  |  |  | 3 |  |  |  |  |  |  |  | 1 |
| QL |  |  |  |  |  |  | 14 |  |  |  |  |  |  |  |  |  |  |  |  |
| SJL-1 |  |  |  |  |  | 20 |  |  |  |  |  |  |  |  |  |  |  |  |  |
| SJL-2 |  |  |  |  |  | 20 |  |  |  |  |  |  |  |  |  |  |  |  |  |
| SJL-3 |  |  |  |  |  | 6 |  |  | 2 |  |  |  |  |  |  | 4 |  |  |  |
| SJL-4 |  |  |  |  |  | 12 |  |  |  |  |  |  |  |  |  |  |  |  |  |
| DF |  |  |  |  |  |  |  |  |  |  |  |  |  | 16 |  |  | 2 |  |  |
| MEK |  |  |  |  |  |  |  |  |  |  |  |  |  | 15 |  |  |  |  |  |
| MuL |  |  |  |  |  |  |  |  |  |  |  |  |  | 10 |  |  |  |  |  |
| XC-1 |  |  |  |  |  |  |  |  |  |  |  |  | 2 | 7 |  |  |  |  |  |
| ZD |  | 2 |  |  |  |  |  |  |  |  |  |  |  | 9 |  |  |  |  |  |
| HS-1 |  |  |  |  |  |  |  |  |  |  |  |  |  | 13 |  |  |  |  |  |
| HS-2 |  |  |  |  |  |  |  |  |  |  |  |  |  | 13 |  |  |  |  |  |
