# Supplementary material for: Climate Is Not All: Evidence From Phylogeography of Rhodiola fastigiata (Crassulaceae) and Comparison to Its Closest Relatives
Source: Front Plant Sci. 2018 Apr 10;9:462. doi: 10.3389/fpls.2018.00462 (PMC5912201; doi:10.3389/fpls.2018.00462)
Supplement: TABLE S4 — Bioclimatic variables (named BIO1 to BIO19) from WorldClim (Hijmans et al., 2005). Variables marked with an asterisk (∗) were used for the climatic niche models of Rhodiola fastigiata. [file Table_4.DOCX]

**Table S4.** Bioclimatic variables (named BIO1 to BIO19) from WorldClim (Hijmans et al., 2005). Variables marked with an asterisk (*) were used for the climatic niche models of *Rhodiola fastigiata*.

| Bioclimatic variables | |
| --- | --- |
| BIO1 | Mean annual temperature |
| BIO2 | Mean diurnal range (mean of monthly (max temp—min temp)) |
| BIO3* | Isothermality (BIO2/BIO7) |
| BIO4 | Temperature seasonality (standard deviation) |
| BIO5* | Max temperature of warmest month |
| BIO6 | Min temperature of coldest month |
| BIO7* | Temperature annual range (BIO5–BIO6) |
| BIO8 | Mean temperature of wettest quarter |
| BIO9 | Mean temperature of driest quarter |
| BIO10 | Mean temperature of warmest quarter |
| BIO11 | Mean temperature of coldest quarter |
| BIO12 | Annual precipitation |
| BIO13* | Precipitation of wettest month |
| BIO14 | Precipitation of driest month |
| BIO15* | Precipitation seasonality (coefficient of variation) |
| BIO16 | Precipitation of wettest quarter |
| BIO17* | Precipitation of driest quarter |
| BIO18 | Precipitation of warmest quarter |
| BIO19 | Precipitation of coldest quarter |
